# Supplementary material for: A cell-free assay implicates a role of sphingomyelin and cholesterol in STING phosphorylation
Source: Sci Rep. 2021 Jun 7;11:11996. doi: 10.1038/s41598-021-91562-z (PMC8184970; doi:10.1038/s41598-021-91562-z)
Supplement: Supplementary file 1 — Supplementary Figures. [file 41598_2021_91562_MOESM1_ESM.pdf]

Supplementary Figure 1

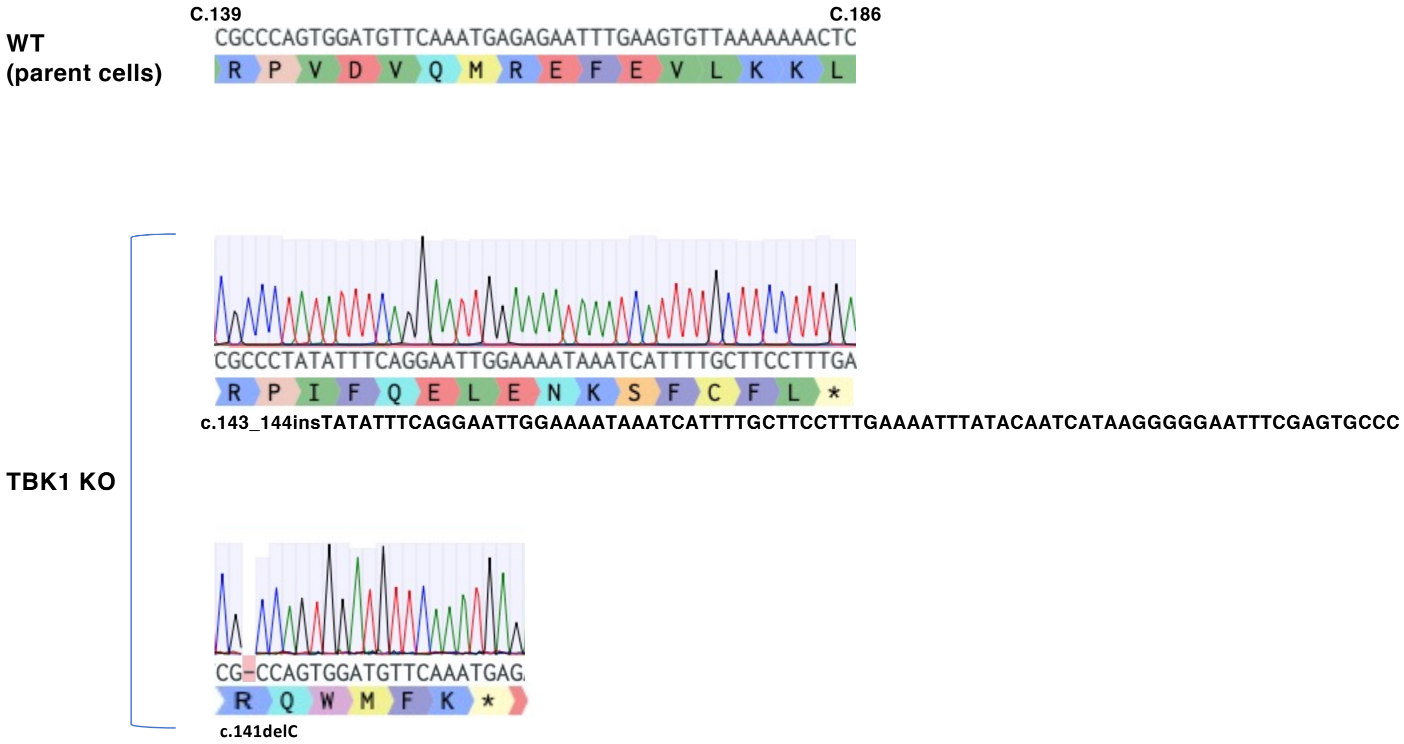

Supplementary Figure 1 | Genomic sequence of TBK1 in TBK1-KO MEFs.
Chromatograms of the exon 3 sequence of TBK1 in TBK1-KO MEFs are shown. Deletion and insertion are indicated under the chromatograms.

Supplementary Figure 2

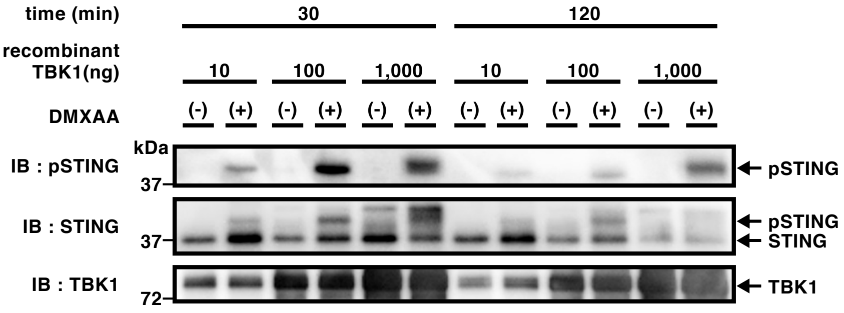

Supplementary Figure 2 | Supplementary materials related to Fig. 2.
TBK1-KO MEFs were stimulated with DMXAA (25 µg/ml) for 0 or 1 h, homogenized in isotonic buffer, and centrifuged at 3,000 x *g* for 5 min. The resulting post-nuclear supernatants were then centrifuged at 100,000 x *g* for 1 h, and the pellets were resuspended in isotonic buffer. The resuspended membrane fractions were incubated with ATP and recombinant TBK1 (10 ng, 100 ng, or 1,000 ng) at 37 °C for 30 or 120 min.

Supplementary Figure 3

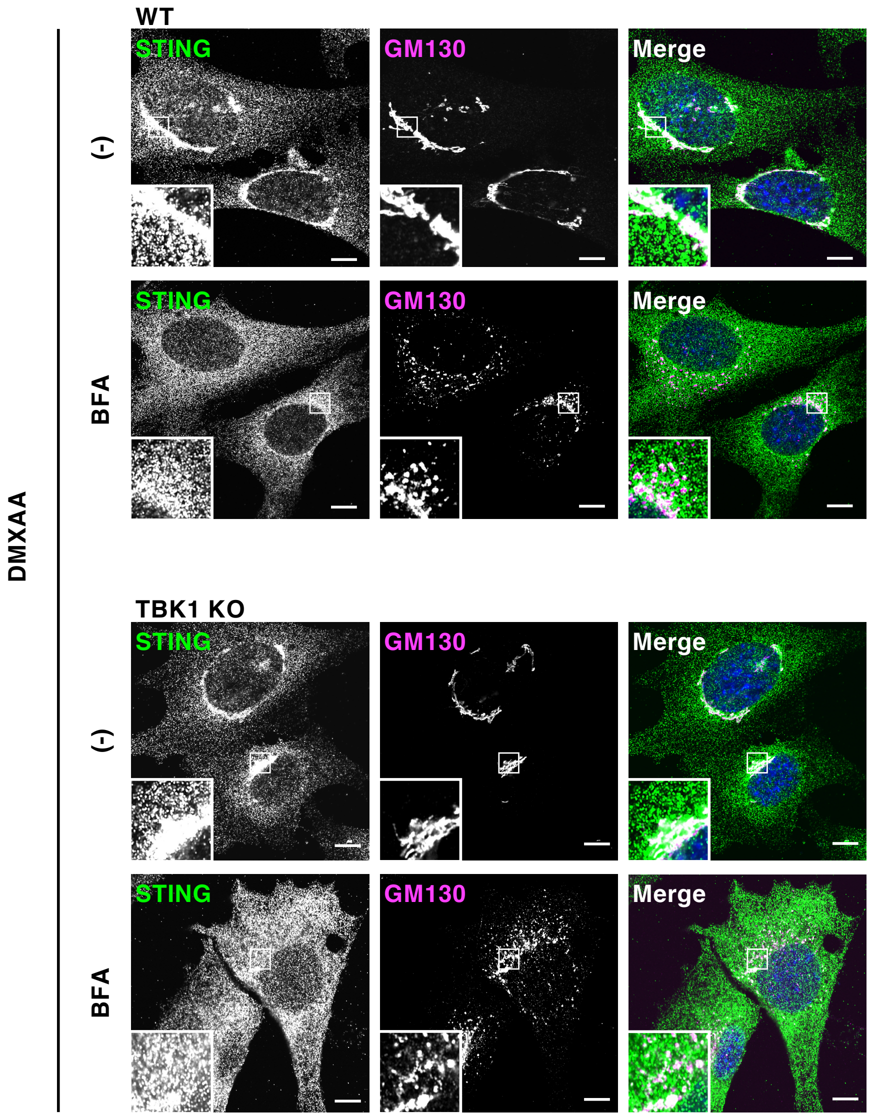

Supplementary Figure 3 | Supplementary materials related to Fig. 3a.
WT or TBK1-KO MEFs were stimulated with DMXAA for 1 h in the presence or absence of BFA (3 µg/mL).

Supplementary Figure 4

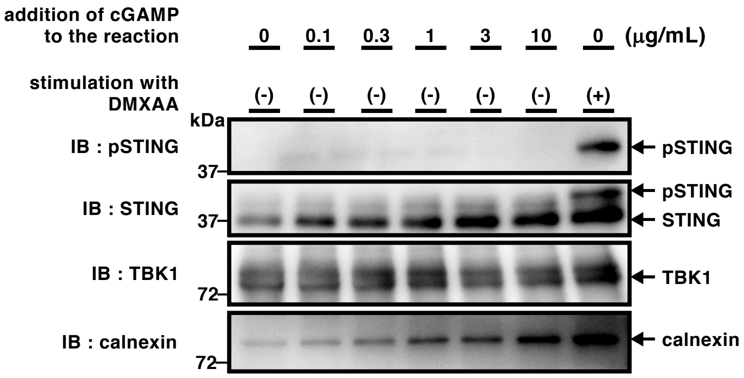

Supplementary Figure 3 | Supplementary materials related to Fig. 3b.
Post-nuclear supernatants of unstimulated TBK1-KO MEFs were centrifuged at 100,000 x *g*. The resulting membrane fraction was resuspended and incubated with recombinant TBK1 and ATP in the presence of 2'3'-cGAMP (0.1 μg/mL - 10 μg/mL) at 37 °C for 30 min. Phosphorylation of STING at Ser365 was examined by western blot. Microsomal membrane fraction prepared from DMXAA-stimulated cells was used as a positive control for the reaction.

Supplementary Figure 5

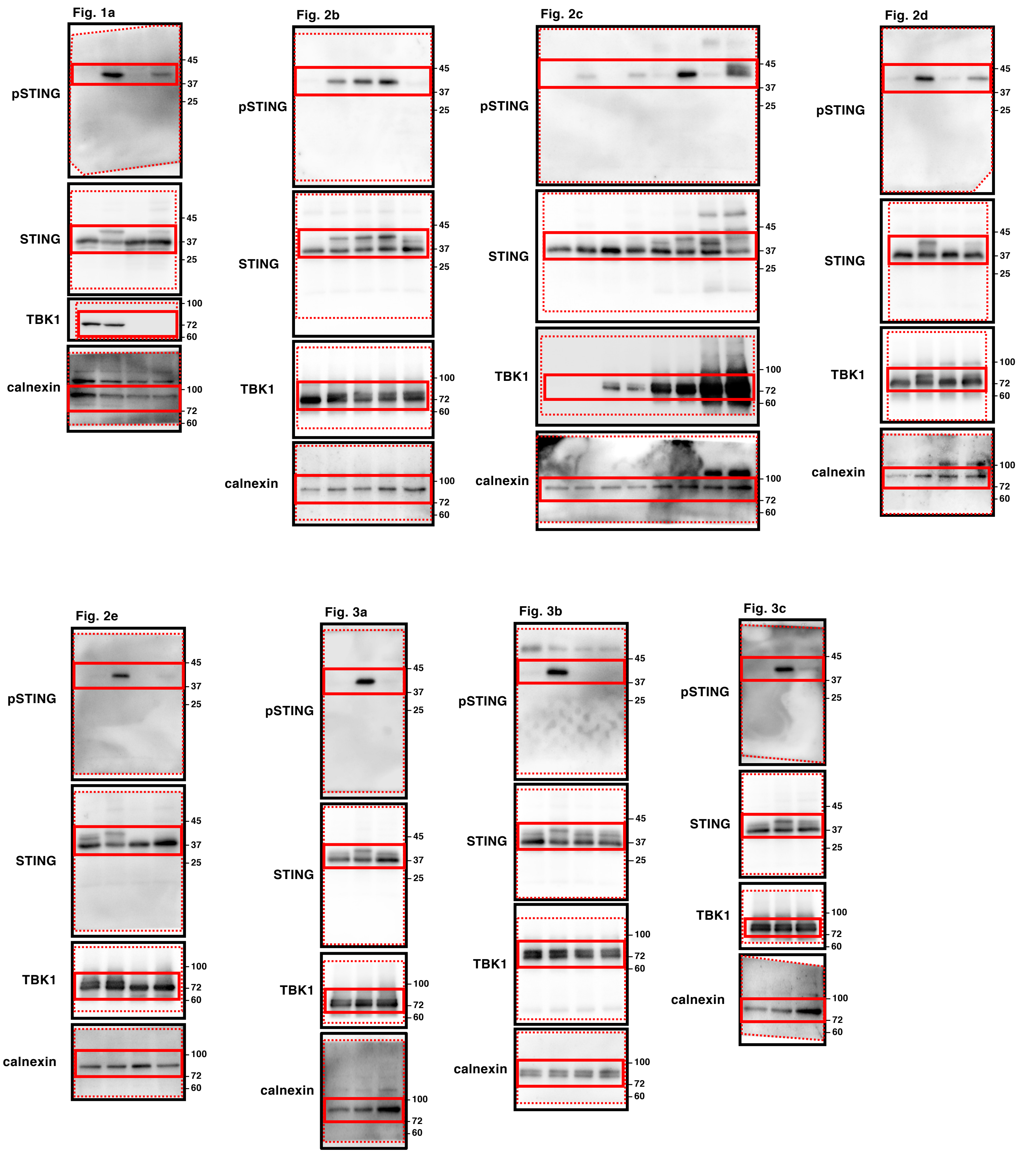

Supplementary Figure 5 | Original western blot images used in this study.
Dotted red lines indicate the edges of the membranes. The individual area bounded by solid red lines are cropped and shown in the corresponding figures.

Supplementary Figure 5 (continued)

Fig. 4a

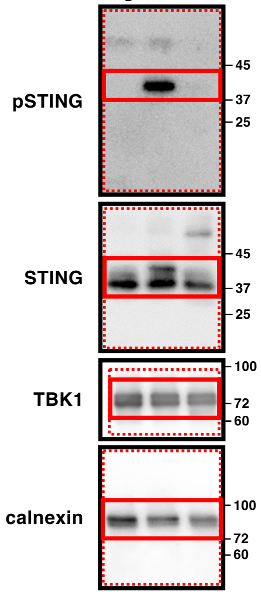

Fig. 4b

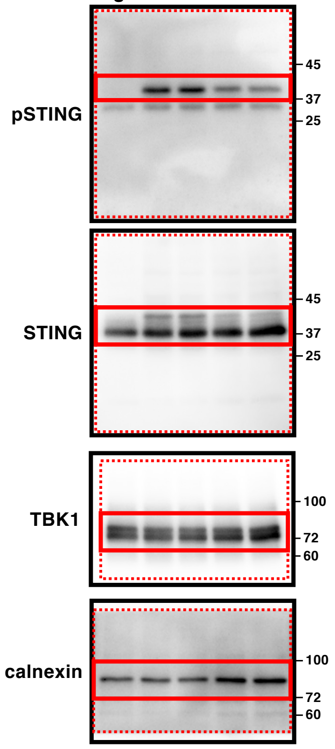

Fig. S2

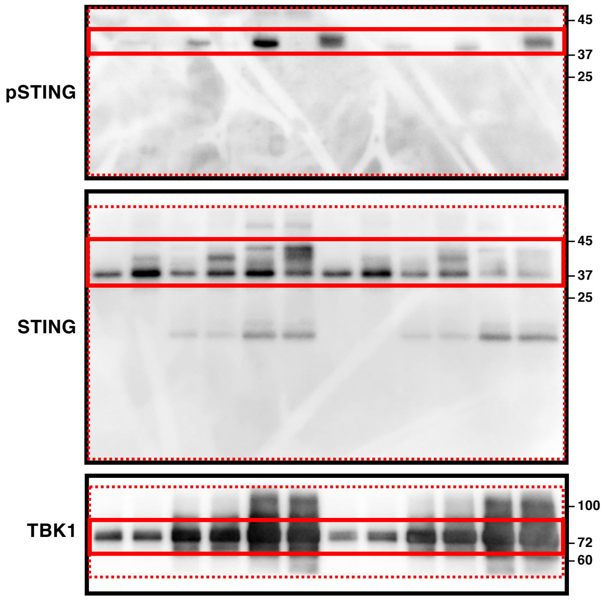

Fig. S4

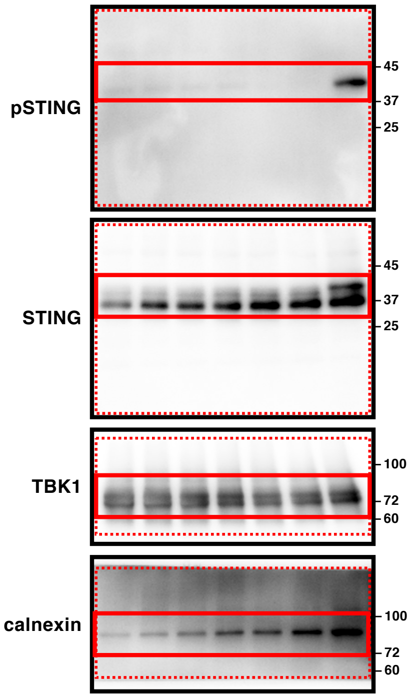

Supplementary Figure 5 (continued) | Original western blot images used in this study. Dotted red lines indicate the edges of the membranes. The individual area bounded by solid red lines are cropped and shown in the corresponding figures.
